# Supplementary material for: Calcium dynamics in habenular astrocytes regulate active coping within behavioral transitions
Source: Commun Biol. 2025 Jul 22;8:1087. doi: 10.1038/s42003-025-08535-5 (PMC12284069; doi:10.1038/s42003-025-08535-5)
Supplement: Supplementary file 1 — Supplementary Information [file 42003_2025_8535_MOESM1_ESM.pdf]

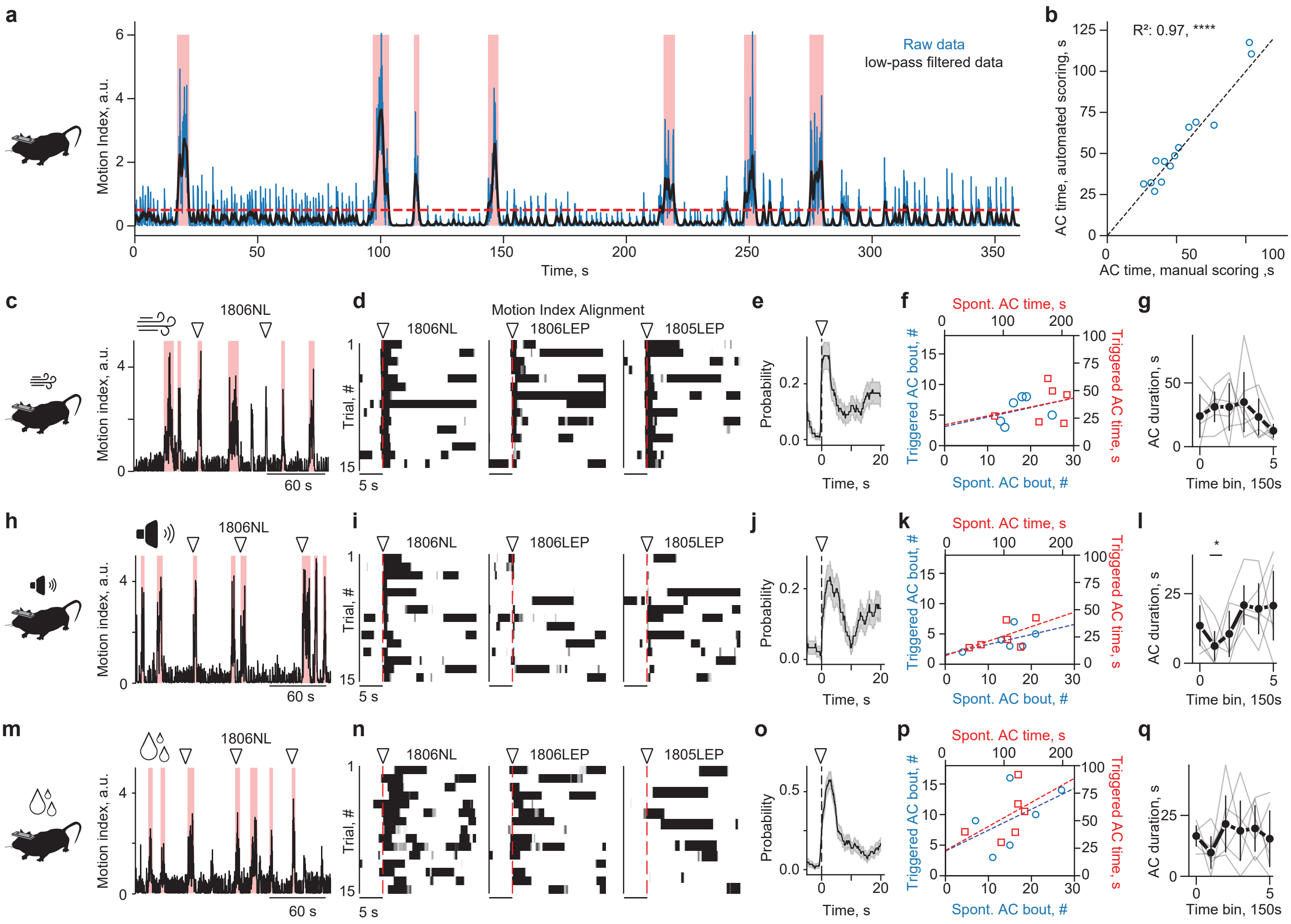

a
Raw data
low-pass filtered data
Motion Index, a.u.
Time, s
b
R²: 0.97, ****
AC time, automated scoring, s
AC time, manual scoring ,s
c
1806NL
Motion index, a.u.
60 s
d
Motion Index Alignment
1806NL
1806LEP
1805LEP
Trial, #
5 s
e
Probability
Time, s
f
Spont. AC time, s
Triggered AC bout, #
Triggered AC time, s
Spont. AC bout, #
g
AC duration, s
Time bin, 150s
h
1806NL
Motion index, a.u.
60 s
i
1806NL
1806LEP
1805LEP
Trial, #
5 s
j
Probability
Time, s
k
Spont. AC time, s
Triggered AC bout, #
Triggered AC time, s
Spont. AC bout, #
l
AC duration, s
Time bin, 150s
m
1806NL
Motion index, a.u.
60 s
n
1806NL
1806LEP
1805LEP
Trial, #
5 s
o
Probability
Time, s
p
Spont. AC time, s
Triggered AC bout, #
Triggered AC time, s
Spont. AC bout, #
q
AC duration, s
Time bin, 150s

Supplementary Fig. 1 Active–to–passive behavioral state transitions in mice. a, Example motion index trace of single mouse, raw trace is shown in blue, low-pass filtered trace (0.5Hz cut-off) is shown in black, dotted red line shows the 0.5 motion index threshold, light red squares show events of AC.

b, Correlation of AC time manually scored against automated analysis, n=14 animals, black dashed line shows regression line, R^2=0.97 ****p<0.0000

c, Sample trace of AC to PC transition when the mouse received airpuffs (arrowheads). Light-red squares show AC.

d, AC bouts timeline aligned with airpuff throughout the 15 trials in three example mice

e, Average probability density of triggering PC to AC transitions upon airpuff (n=6 mice).

f, Correlation of spontaneous AC time against triggered AC time (n=6 animals, red squares) and spontaneous AC bout counts against triggered AC bout counts (n=6 animals, blue circles). Dashed lines show regression lines for AC time (red) R^2=0.09 p=0.57 and AC bout count (blue) R^2=0.10 p=0.54.

g, Time-binned population-average spontaneous AC duration throughout the airpuff task (n=6 mice; bin = 150s), ANOVA $F_{5, 25}$ = 2.09 p=0.10.

h, Same as c, but for the aversive sound.

i, Same as d, but for the aversive sound.

j, Same as e, but for the aversive sound (n=6 mice).

k, Same as f, but for the aversive sound. Dashed lines show regression lines for AC time (red) R^2=0.37 p=0.20 and AC bout counts (blue) R^2=0.29 p=0.27.

l, Same as f, but for the aversive sound (n=6 mice), ANOVA $F_{5, 25}$ = 2.14 *p=0.09.

m, Same as c, but for water drops (n=6 mice).

n, Same as d, but for water drops.

o, Same as e, but for water drops (n=6 mice).

p, Same as f, but for water drops (n=6 mice). Dashed lines show regression lines for AC time (red) R^2=0.25 p=0.32 and AC bout counts (blue) R^2=0.24 p=0.33.

q, Same as g, but for the water drops, ANOVA $F_{5, 25}$ = 1.28 p=0.30.

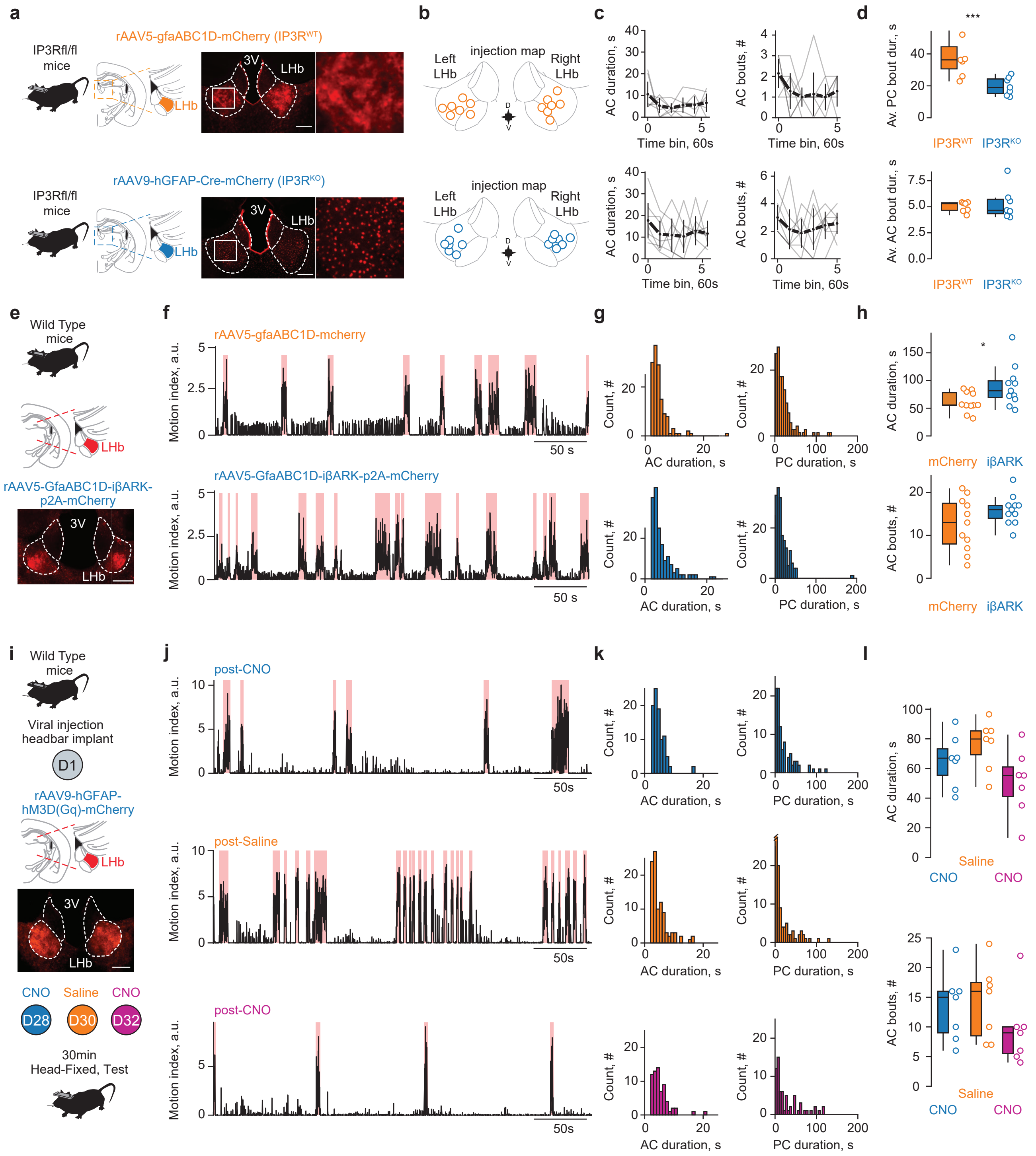

a
IP3Rfl/fl mice
rAAV5-gfaABC1D-mCherry (IP3R^WT)
3V
LHb
rAAV9-hGFAP-Cre-mCherry (IP3R^KO)
b
injection map
Left LHb
Right LHb
D
V
c
AC duration, s
Time bin, 60s
AC bouts, #
d
Av. PC bout dur., s
***
IP3R^WT
IP3R^KO
Av. AC bout dur., s
e
Wild Type mice
rAAV5-GfaABC1D-iβARK-p2A-mCherry
f
rAAV5-gfaABC1D-mcherry
Motion index, a.u.
50 s
rAAV5-GfaABC1D-iβARK-p2A-mCherry
g
Count, #
AC duration, s
PC duration, s
h
AC duration, s
*
mCherry
iβARK
AC bouts, #
i
Wild Type mice
Viral injection headbar implant
D1
rAAV9-hGFAP-hM3D(Gq)-mCherry
CNO
Saline
D28
D30
D32
30min Head-Fixed, Test
j
post-CNO
post-Saline
50s
k
l

Supplementary Fig. 2 Habenular astrocytes temporally regulates coping behaviors.

a, Left: mouse $IP3R^{fl/fl}$ line used. Middle top: viral construct and strategy for control mice injected with mCherry, $IP3R^{WT}$. Middle down, viral construct and strategy for $IP3R^{KO}$ mice injected with Cre, $IP3R^{KO}$. Right top: representative image of bilateral mCherry injection, white square shows the area for higher magnification scale bar = 200 µm. Right bottom: representative image of bilateral Cre injection, white square shows the area for higher magnification, scale bar = 200 µm.

b, Schematic injection map for bilateral injection in LHb in $IP3R^{WT}$ (Top) and $IP3R^{KO}$ (bottom), D, dorsal; V, ventral.

c, Left: Time-binned population-average AC duration throughout the head-fixed task (bin = 1 min) in $IP3R^{WT}$ animals (Top, n=7 animals, ANOVA $F_{5,30}$ = 1.2 p=0.29) and $IP3R^{KO}$ animals (Bottom, n=7 animals, ANOVA $F_{5,30}$ = 0.7 p=0.616). Right: same as left but for AC bout counts in $IP3R^{WT}$ animals (Top, n=7 animals, ANOVA $F_{5,30}$ = 0.312 p=0.88) and $IP3R^{KO}$ animals (Bottom, n=7 animals, ANOVA $F_{5,30}$ = 0.846 p=0.53) **d**, Top, average PC bout duration in $IP3R^{WT}$ (n=7, orange) and $IP3R^{KO}$ animals (n=7, blue). Data are presented as box plot, min to max, median and scatter. Two-sided t-test, t_{12}=3.58 ***p=0.004. Bottom, average AC bout duration in same groups. Data are presented in the same way. Two-sided t-test, t_{12}=0.51 p=0.62.

e, Top: schematic of experimental strategy. Bottom: representative image of rAAV5-GfaABC1D- iβARK-p2A-mCherry bilateral LHb expression in WT mice; scale bar, 200 µm.

f, Sample trace of AC to PC transitions in a mouse virally injected with a control rAAV5-gfaABC1D-mCherry virus (top), or a rAAV5-GfaABC1D- iβARK-p2A-mCherry virus (bottom). Light-orange squares indicate AC.

g, Histogram of all counted AC bouts duration (left) and PC bouts duration (right) in control virus (top, orange) and iβARK–injected (bottom, blue) (n=11 mice/group).

h, Total AC duration (top) and AC bouts number (bottom). Data are presented as box plot, min to max, median and scatter in orange for mCherry and blue for iβARK–injected mice. Two-sided unpaired t-test, $t_{14,579}$=2.33 *p=0.035 for total AC duration and $t_{15.612}$=1.581 p=0.134 for number of AC bouts.

i, Top: schematic of experimental strategy. Middle: representative image of rAAV9-hGFAP- hM3D(Gq)-mCherry bilateral LHb expression in WT mice; scale bar, 200 µm. Bottom : Experimental settings for injected mice.

j, Sample trace of AC to PC transitions in a mouse virally injected with a rAAV9-hGFAP- hM3D(Gq)-mCherry virus in post-CNO condition (top), post-Saline (middle) and post-CNO again (bottom). Light-orange squares indicate AC.

k, Histogram of all counted AC bouts duration (left) and PC bouts duration (right) in hM3D(Gq)–injected mice post-CNO (top, blue), post-Saline (middle, orange) and post-CNO (bottom, magenta) (n=7 mice).

l, Total AC duration (top) and bouts number (bottom) for hM3D(Gq)–injected mice. Data are presented as box plot, min to max, median and scatter in blue for post-CNO, orange for post-Saline and magenta for post-CNO sessions for total AC duration (top, ANOVA $F_{2,\ 12}$ = 3.320 p = 0.071) and number of AC bouts (bottom, ANOVA $F_{2,\ 12}$ = 0.875 p = 0.442).

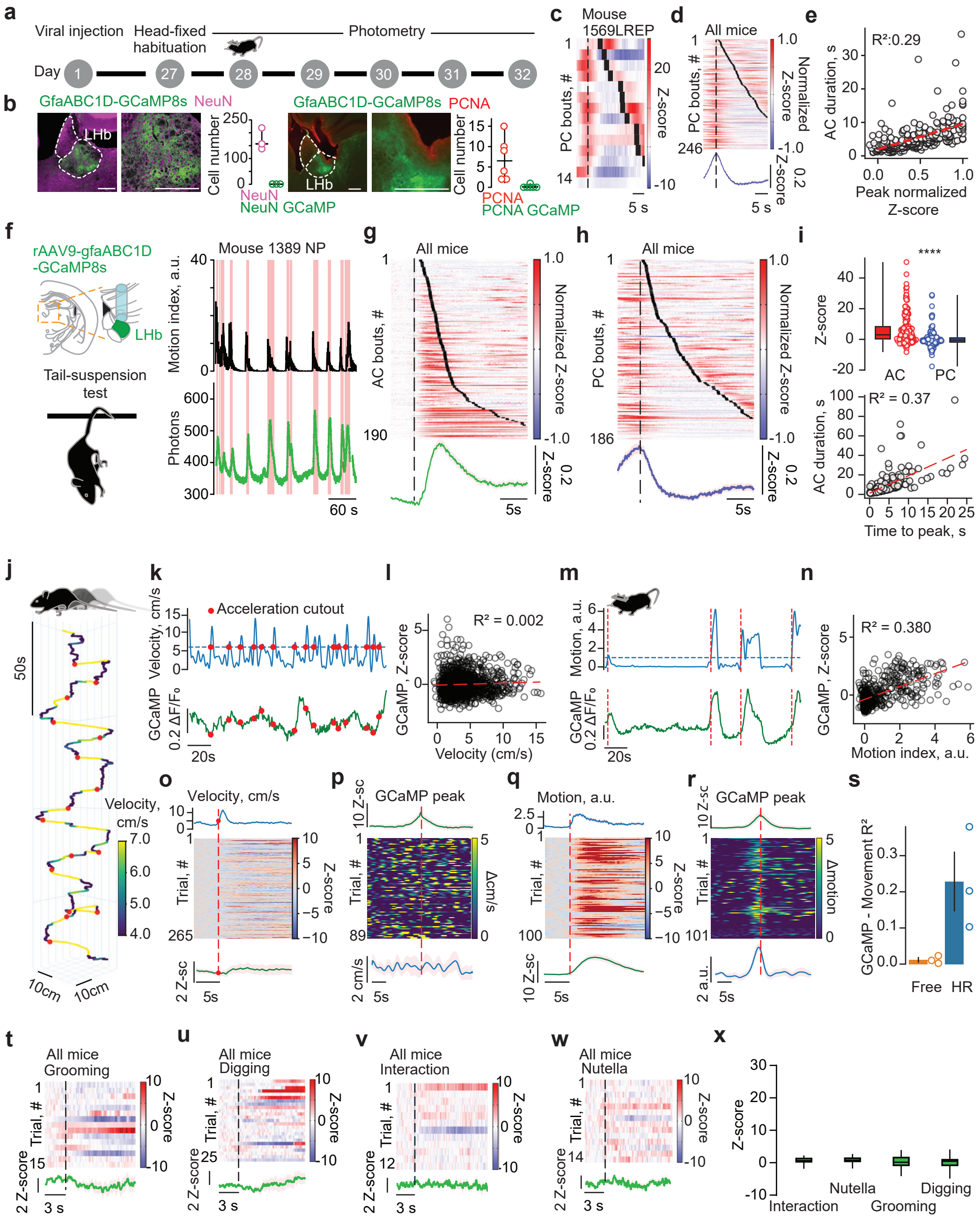
a
Viral injection
Head-fixed habituation
Photometry
Day 1 27 28 29 30 31 32
b
GfaABC1D-GCaMP8s NeuN
LHb
Cell number
250
100
0
NeuN
NeuN GCaMP
GfaABC1D-GCaMP8s PCNA
LHb
15
10
5
0
PCNA
PCNA GCaMP
c
Mouse 1569LREP
PC bouts, #
1
14
20
Z-score
-10
5 s
d
All mice
PC bouts, #
1
246
1.0
Normalized Z-score
-1.0
0.2 Z-score
5 s
e
R²:0.29
AC duration, s
30
20
10
0
0.0
0.5
1.0
Peak normalized Z-score
f
rAAV9-gfaABC1D -GCaMP8s
LHb
Tail-suspension test
Mouse 1389 NP
Motion index, a.u.
40
0
Photons
600
500
400
300
60 s
g
All mice
AC bouts, #
1
190
1.0
Normalized Z-score
-1.0
0.2 Z-score
5s
h
All mice
PC bouts, #
1
186
1.0
Normalized Z-score
-1.0
0.2 Z-score
5s
i
****
Z-score
40
20
0
-20
AC
PC
R² = 0.37
AC duration, s
100
80
60
40
20
0
0 5 10 15 20 25
Time to peak, s
j
50s
Velocity, cm/s
7.0
6.0
5.0
4.0
10cm
10cm
k
Acceleration cutout
Velocity, cm/s
15
10
5
0
GCaMP 0.2 ΔF/F₀
20s
l
R² = 0.002
GCaMP, Z-score
5
0
0 5 10 15
Velocity (cm/s)
m
Motion, a.u.
6
4
2
0
GCaMP 0.2 ΔF/F₀
20s
n
R² = 0.380
GCaMP, Z-score
5
0
0 2 4 6
Motion index, a.u.
o
Velocity, cm/s
10
0
1
Trial, #
265
10
5
0
−5
−10
Z-score
2 Z-sc
5s
p
GCaMP peak
10 Z-sc
1
Trial, #
89
5
0
Δcm/s
2 cm/s
5s
q
Motion, a.u.
2.5
0
1
Trial, #
100
10
5
0
−5
−10
Z-score
10 Z-sc
5s
r
GCaMP peak
10 Z-sc
1
Trial, #
101
5
0
Δmotion
2 a.u.
5s
s
GCaMP - Movement R²
0.3
0.2
0.1
0.0
Free
HR
t
All mice Grooming
1
15
Trial, #
10
0
-10
Z-score
2 Z-score
3 s
u
All mice Digging
1
25
Trial, #
10
0
-10
Z-score
2 Z-score
3 s
v
All mice Interaction
1
12
Trial, #
10
0
-10
Z-score
2 Z-score
3 s
w
All mice Nutella
1
14
Trial, #
10
0
-10
Z-score
2 Z-score
3 s
x
Z-score
30
20
10
0
-10
Interaction
Nutella
Grooming
Digging

Supplementary Fig. 3 LHb Astrocyte dynamics during active coping states.

a, Schematic of experimental design for photometry experiments.

b, Left panel, representative image of rAAV9-GfaABC1D-GCaMP8s expression (green) counterstained with an anti-NeuN (magenta) with two magnifications (scale bar, 200 µm) and counted cell number of $NeuN^+$ (magenta) and $NeuN^+GCaMP^+$ (green) in LHb. Right panel: representative image of rAAV9-GfaABC1D-GCaMP8s expression (green) counterstained with an anti-PCNA (red) with two magnifications (scale bar, 200 µm) and cell count of $PCNA^+$ (red) and $PCNA^+GCaMP^+$ (green) cells in LHb.

c, Sample heatmap related to photometry signal across trials. Data are presented as a heatmap of Z-score for each PC bout, sorted from shortest to longest, dashed line shows onset of AC while thick black lines show offset.

d, Top: heatmap of Z-score for each PC bout, normalized per animal (n=1 animals, 246 trials), sorted and represented as c. Bottom: aligned averaged and normalized Z-score trace for all PC bouts.

e, Calcium maximum peak in function of AC bout duration, red dashed line shows regression line, R^2=0.29, ****p<0.0001.

f, Left panel, schematic of experimental design for photometry experiment. Right panel, sample motion index trace during the TST from single mouse and aligned raw photometric signal in green. Light-red squares indicate AC.

g, Top: heatmap of Z-score for each AC bout, normalized per animal (n=13 animals, 190 trials), sorted and represented as c. Bottom: aligned averaged and normalized Z-score trace for all AC bouts.

h, Top: heatmap of Z-score for each PC bout, normalized per animal (n=13 animals, 186 trials), sorted and represented as c. Bottom: aligned averaged and normalized Z-score trace for all PC bouts.

i, Top: average Z-score for the last second of AC and PC bout. Data are presented as box plot, min to max, median and scatter. Two-tailed Wilcoxon signed rank test, ****p<0.0001. Bottom: calcium rising time in function of AC bout duration, red dashed line shows regression line, R^2=0.37 ****p<0.0001.

j, Sample 3D trace of a freely moving mice. Trace accounts for the x, y coordinate of the mouse centroid inside a phenotyper and the velocity was color coded. Red dots indicate detected acceleration events (Velocity > 6cm/s).

k, Sample velocity trace (top) of a freely moving mice, aligned to the GCaMP signal trace (bottom). Red dots indicate detected acceleration events (Velocity > 6cm/s).

l, Example of correlation between velocity and GCaMP signal in a freely moving mice. R^2=0.002 p = 0.098

m, Sample motion trace (top) of a head-fixed mice, aligned to the GCaMP signal trace (bottom). Dashed red lines represents detected AC bout onsets (>1 a.u.).

n, Example of correlation between motion and GCaMP signal in a head-fixed mice. R^2=0.38 p**** < 0.0001.

o, Top, average velocity trace, aligned to the detected acceleration events (red dots). Middle, heatmap of GCaMP signal Z-score aligned to acceleration (N=3 animals, n=265 events). Bottom, trial-average GCaMP signal z-score aligned to acceleration events.

p, Top, average GCaMP trace aligned to the detected Ca^{2+} event peaks (red dashed line). Middle, heatmap of velocity aligned to GCaMP peaks (N=3 animals, n=89 events). Bottom, event-average velocity trace aligned to GCaMP peaks.

q, Top, average motion trace, aligned to the detected AC bout onsets (red dashed line). Middle, heatmap of GCaMP signal z-score aligned to AC (N=3 animals, n=100 events). Bottom, event-average GCaMP signal z-score aligned to AC bout onsets.

r, Top, average GCaMP trace aligned to the detected Ca^{2+} event peaks (red dashed line). Middle, heatmap of motion aligned to GCaMP peaks (N=3 animals, n=101 events). Bottom, event-average motion trace aligned with GCaMP peaks.

s, R^2 for GCaMP signal and movement (velocity/motion) in freely moving (orange) and head-fixed (Blue) condition. Data are presented as bar plot, mean±sem and scatter (n=3 animals).

t, Heatmap of Z-score for each grooming event (n=4 animals, 15 trials), onset of behavior is represented by the black dashed lines and aligned with the average trace.

u, Heatmap of Z-score for each digging event (n=4 animals, 25 trials), onset of behavior is represented by the black dashed lines and aligned with the average trace.

v, Heatmap of Z-score for each interaction event (n=4 animals, 12 trials), onset of behavior is represented by the black dashed lines and aligned with the average trace.

w, Heatmap of Z-score for each licking event (n=4 animals, 14 trials), onset of behavior is represented by the black dashed lines and aligned with the average trace.

x, Average Z-score of 3 seconds post-onset of behavior. Data are presented as box plot, min to max, median. Paired two-sided t-test of the average Z-score 3s post stimuli

and 3s baseline, grooming t_{14}=0.99 p=0.3383, digging t_{24}=0.92 p=0.3682, interaction t_{11}=0.35 p=0.7315 and nutella t_{13}=0.89 p=0.3872.

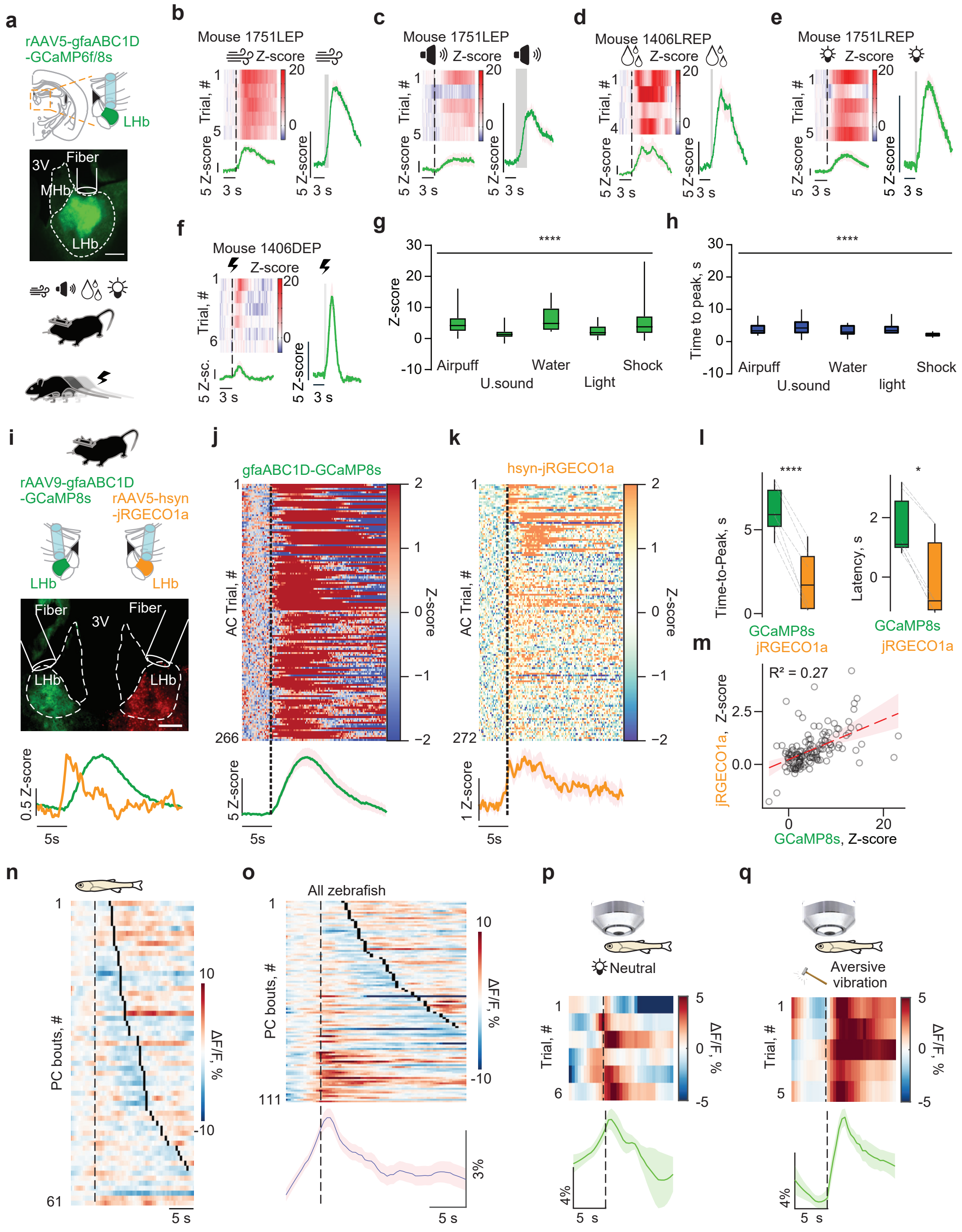

a
rAAV5-gfaABC1D
-GCaMP6f/8s
LHb
3V
Fiber
MHb
LHb
b
Mouse 1751LEP
Z-score
Trial, #
5 Z-score
3 s
c
Mouse 1751LEP
d
Mouse 1406LREP
e
Mouse 1751LREP
f
Mouse 1406DEP
g
Z-score
Airpuff
U.sound
Water
Light
Shock
****
h
Time to peak, s
Airpuff
U.sound
Water
light
Shock
****
i
rAAV9-gfaABC1D
-GCaMP8s
rAAV5-hsyn
-jRGECO1a
LHb
Fiber
3V
0.5 Z-score
5s
j
gfaABC1D-GCaMP8s
AC Trial, #
Z-score
k
hsyn-jRGECO1a
l
Time-to-Peak, s
Latency, s
GCaMP8s
jRGECO1a
m
R² = 0.27
jRGECO1a, Z-score
GCaMP8s, Z-score
n
PC bouts, #
ΔF/F, %
5 s
o
All zebrafish
3%
p
Neutral
Trial, #
4%
5 s
q
Aversive
vibration

Supplementary Fig. 4 LHb Astrocyte dynamics during active coping states.

a, Top, Schematic depicting LHb viral injection and optic fiber placement. Middle, representative image of rAAV9-GfaABC1D-GCaMP8s expression and fiber track in LHb (middle). 3V: Third ventricle; MHb: medial habenula. Bottom : experimental plan for photometry recordings under various aversive stimuli.

b, Left: sample photometry signal from a single mouse exposed to airpuff showing heatmap of Z-score and average Z-score trace (airpuff is indicated by black dashed line). Right: average trace for all mice (n=18).

c, Same as b, but for an aversive sound (n=12 mice).

d, Same as b, but for water drops (n=6 mice).

e, Same as b, but for an aversive light (n=11 mice).

f, Same as b, but for footshocks, in freely moving mice (n=6 mice).

g, Average Z-score of 3 seconds post-stimuli. Data are presented as box plot, min to max, median. Paired two-sided t-test of the average Z-score 3s post stimuli and 3s baseline, airpuff t_{54}=9.861 ****p<0.0001, sound t_{65}=8.262 ****p<0.0001, water drop t_{14}=5.379 ****p<0.0001, light t_{60}=9.876 ****p<0.0001, shock t_{35}=5.972 ****p<0.0001. ANOVA $KW_{5, 233}$ =58.56 ****p<0.0001.

h, Average time to peak post stimuli exposition. Data are presented as in g. ANOVA $KW_{5, 233}$ =48.75 ****p<0.0001.

i, Top, Schematic depicting LHb viral injection and optic fiber placement. Middle, representative image of combined rAAV9-GfaABC1D-GCamp8s and fiber track in the left LHb with rAAV5-hsyn-jRGECO1a and fiber track in the right LHb. 3V: Third ventricle. Bottom : Trial-averaged GCaMP (green) and jRGECO1 (orange) z-score responses to AC onset in one representative animal .

j, Top, heatmap of z-scored GCaMP calcium activity aligned to AC bout, (n=3 mice, 266 trials). Bottom, trial-average GCaMP signal z-score aligned to the onset of AC bout.

k, Top, heatmap of z-scored jRGECO1a calcium activity aligned to AC bout, (n=3 mice, 272 trials). Bottom, average jRGECO1a signal z-score aligned to the onset of AC bout.

l, Left, Time-to-peak from an AC bout onset for GCaMP and jRGECO1a signal. Paired two-sided t-test t_5=10.45 ****p<0.0001. Right, signal latency from an AC bout onset for GCaMP and jRGECO1a signal. Two-tailed Wilcoxon signed rank test, *p=0.031.

m, jRGECO1a response to AC bout onset in function of GCaMP8s response to AC bout onset, red dashed line shows regression line, R^2=0.27, ****p<0.0001.
n, Heatmap of astroglial calcium traces during PC bouts from an example fish. Warm colors represent increased activity, cold colors represent decreased activity. The dashed line indicates PC bout onset, and the thick black line represents PC bout offset.
o, Top : Heatmap of astroglial calcium traces during PC bouts from all recorded fish (*n*=5). Bottom : average of all PC bouts from all recorded fish (n=5).**p,** Top**:** Illustration of a head-restrained, awake, behaving juvenile zebrafish under a two-photon microscope facing a neutral light. Middle: Astroglial calcium responses to consecutive light stimulations. Bottom, Trial-averaged astroglial light responses from an example animal.
q, Top: Illustration of a head-restrained, awake, behaving juvenile zebrafish under a two-photon microscope facing an aversive vibration stimulus.
Middle: Astroglial calcium responses to consecutive vibrations. Bottom: Trial-averaged astroglial light responses from an example animal.

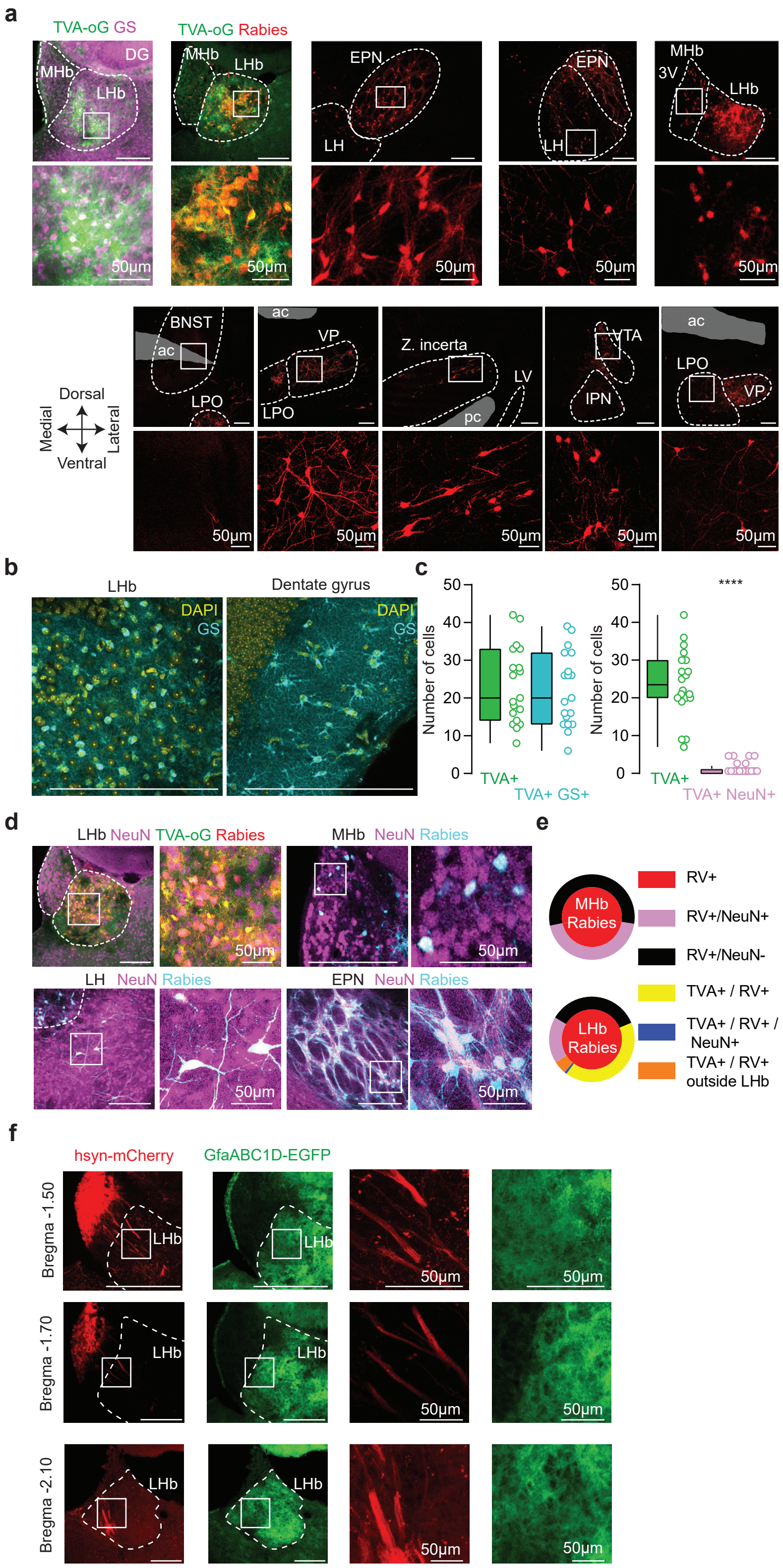

a
TVA-oG GS
TVA-oG Rabies
DG
MHb
LHb
EPN
LH
3V
BNST
ac
LPO
VP
Z. incerta
LV
pc
VTA
IPN
Dorsal
Medial
Lateral
Ventral
50µm
b
LHb
Dentate gyrus
DAPI
GS
c
Number of cells
TVA+
TVA+ GS+
****
TVA+ NeuN+
d
LHb NeuN TVA-oG Rabies
MHb NeuN Rabies
LH NeuN Rabies
EPN NeuN Rabies
e
MHb Rabies
LHb Rabies
RV+
RV+/NeuN+
RV+/NeuN-
TVA+ / RV+
TVA+ / RV+ / NeuN+
TVA+ / RV+ outside LHb
f
hsyn-mCherry
GfaABC1D-EGFP
Bregma -1.50
Bregma -1.70
Bregma -2.10

Supplementary Fig. 5 Input organization of LHb astrocytes.

a. Representative images depicting LHb injection site for rAAV5-GFAP-TVA-oG (green) counterstained with an anti-Glutamine synthetase (GS) antibody, after ∆G-Rabies-mCherry (red) injection and brain regions with positive rabies positive cells, the image below shows a higher magnification depicted by the white square. Respective scale bars: 200 µm and 50 µm. 3V, third ventricle; ac, anterior commissure; BNST, bed nucleus stria terminalis EPN, entopeduncular nucleus; IPN, interpeduncular nucleus; LH, lateral hypothalamus; LPO, lateral preoptic area; LV, lateral ventricle; MHb, medial habenula; pc, posterior commissure; VP, ventral pallidum; VTA, ventral tegmental area.

b, Example high magnification images of the same histological section counterstained with anti-GS (cyan) and DAPI (yellow) from LHb (left) and Dentate Gyrus (right). Scale bars, 200 µm.

c, Left, Cell number of TVA^+ (green) and TVA^+GS^+ (cyan) in LHb. Right, cell number of TVA^+ (green) and TVA^+NeuN^+ (magenta) in LHb Two-tailed Wilcoxon signed rank test, ****p <0.0001. Data are presented as box plot, min to max, median and scatter.

d, Representative images of positive TVA and rabies cells for LHb (green and red), MHb, LH and EPN (cyan) and counterstained with an anti-NeuN (magenta). Corresponding right image shows a higher magnification of the area depicted by the white square. Scale bars, 200 µm and 50 µm.

e, Quantification of neuronal and non-neuronal rabies positive cells in MHb and LHb. Data are presented as pie charts representing RV^+ cells in red, RV^+NeuN^+ in magenta, RV^+NeuN^- cells in black, TVA^+RV^+ in yellow, $TVA^+RV^+NeuN^+$ in blue and TVA^+RV^+outside LHb in orange over total RV^+ cells (n=7 animals).

f, Representative image of AAV1-hsyn-mCherry injection in MHb (red) with axonal projections through astrocytic LHb territory (green) across three bregma position, magnifications are shown on the right and are depicted by the white square (n=1 animal). Scale bars, 200 µm and 50 µm.
