# Supplementary material for: Calcium dynamics in habenular astrocytes regulate active coping within behavioral transitions
Source: Commun Biol. 2025 Jul 22;8:1087. doi: 10.1038/s42003-025-08535-5 (PMC12284069; doi:10.1038/s42003-025-08535-5)
Supplement: Supplementary file 2 — Description of Additional Supplementary Files [file 42003_2025_8535_MOESM2_ESM.doc]

Description of Additional Supplementary Files

File name: Supplementary Video 1

Description: Video representing behavioural transitions
